# Buzz pollination: investigations of pollen expulsion using the discrete element method

**DOI:** 10.1098/rsif.2024.0526

**Published:** 2025-01-22

**Authors:** Caelen Boucher-Bergstedt, Mark Jankauski, Erick Johnson

**Affiliations:** ^1^ Department of Mechanical & Industrial Engineering, Montana State University, Bozeman, MT, USA

**Keywords:** buzz pollination, discrete element method, pollen, anther, vibrations

## Abstract

Buzz pollination involves the release of pollen from, primarily, poricidal anthers through vibrations generated by certain bee species. Despite previous experimental and numerical studies, the intricacies of pollen dynamics within vibrating anthers remain elusive due to the challenges in observing these small-scale, opaque systems. This research employs the discrete element method to simulate the pollen expulsion process in vibrating anthers. By exploring various frequencies and displacement amplitudes, a correlation between how aggressively the anther shakes and the initial rate of pollen expulsion is observed under translating oscillations. This study highlights that while increasing both the frequency and displacement of vibration enhances pollen release, the rate of release does not grow linearly with their increase. Our findings also reveal the significant role of pollen–pollen interactions, which account for upwards of one-third of the total collisions. Comparisons between two types of anther exits suggest that pore size and shape also influence expulsion rates. This research provides a foundation for more comprehensive models that can incorporate additional factors such as cohesion, adhesion and Coulomb forces, paving the way for deeper insights into the mechanics of buzz pollination and its variability across different anther types and vibration parameters.

## 1. Introduction

There is concern that any loss of animal pollinators, both buzzing and non-buzzing, would reduce global food production by about 5–8% and necessitate a much larger increase in agricultural land to meet the demand with non-pollinated crops [1]. Moreover, around 75% of all crops would see a reduction in quality and yield without visiting pollinators [2,3]. These changes would impoverish the nutritional balance of the human diet and disproportionately impact different parts of the world [1,3]. Accounting for approximately 9–10% of all flowering plants [4–6], and visited by around 58% of all bee species [4,7], flowers with poricidal anthers, defined as anthers with an apical pore that require buzzing to extract pollen, include many important crops, such as tomatoes, eggplants and kiwis [4–9]. Given the importance of buzz pollination to our food supply and the sensitivity of crop productivity to the presence of sufficient pollinators [4], alternative pollinating treatments could be considered. However, a recent study of tomatoes (*Solanum lycopersicum* L.) showed a more than doubling of fruit weight when treated by bees that participate in buzz pollination rather than through artificial methods and suggests that more research is needed [10].

The exploration of buzz pollination dates back to at least 1902, with laboratory investigations emerging in the 1970s [11]. After landing on a flower, bees bite the anther and rapidly contract and relax their indirect flight muscles. This ‘buzzing’ produces vibrations that translate and deform the anther, causing pollen to be released [6,12,13]. Studies have revealed that bee-generated buzzes consist of a set of short bursts lasting around 0.5 s, with each individual burst varying from a few milliseconds to 0.1 s [6] and typically ranging in frequency between 100 and 400 Hz across various bee species [6,14]. Exciting anthers with artificially generated vibrations, experiments have demonstrated a direct correlation between vibration amplitude and pollen release, where vibration amplitude has been referred to as peak anther displacement [15], velocity [16,17] and acceleration [14,18].

While frequency has been shown to have an impact on pollen release, its specific contribution has been difficult to decouple from the vibration amplitude. Researchers have hypothesized that plant and bee physiology maximize the release of pollen at specific vibration frequencies, such that the resonance of the anther is excited [12,14,15,17,18], but this has largely not been observed experimentally [8]. Harder and Barclay demonstrate that under constant energy the percentage of comfrey *Symphytum officinale* pollen released only increases above 400 Hz and outside the general range of buzzing [15]. Rosi-Denadai *et al*. vibrated tomato plants *Solanum lycopersicum* L*.* from 100 to 1600 Hz and did not observe a resonant response that significantly improved pollen release [14]. While bumblebees have been observed to change their frequencies [19,20], a recent study shows that this is not necessarily in search of a frequency that is rewarded with the most pollen released [21]. On the other hand, Jankauski *et al.* postulated based on computational modelling that some of the resonant frequencies of *Solanum elaeagnifolium* fell within proximity to reported buzzing frequencies—but only if the mass of the buzz pollinating bee on the anther was considered [12]. This suggests that bees may leverage vibration amplification to increase anther deformation rate, and in turn, pollen expulsion. Consequently, it is possible that physical experiments that neglect bee mass may not observe anther resonance and the corresponding increase in pollen expulsion rates. Still, additional experimental and modelling efforts are needed to identify if there is an ‘optimal’ buzzing frequency for maximal pollen expulsion and what parameters affect this frequency.

One of the largest hurdles in experimental work on buzz pollination is the difficulty to see the internal workings of a flower’s anthers and pollen while vibrating due to the small scale, high speeds and the opacity of the anther wall. Numerical models provide an alternative approach, and efforts have been made to understand buzz pollination via statistical mechanics [22] and the billiards model [23], where single pollen particles reflect off moving, rather than stationary, walls. As such, the focus of inquiry has allowed for a shift from observable, macro-scale phenomena, such as the amount of pollen expelled, towards finer scale processes within the anther [6,14,16,22].

Buchmann & Hurley [22] proposed that the rate of pollen expulsion would increase with an increase of stored kinetic energy in the anther. To capture this process, their model splits the rate of energy change in the system into two parts. The first part theorized that as the anther walls vibrate back and forth, there would be a net increase of energy in the system from pollen–wall and pollen–pollen collisions. The second part suggested that as individual pollen are expelled, they no longer contribute to the total energy, which causes the energy to decrease as they leave. The result of these considerations indicates that there is a maximum available energy that is proportional to the amount of pollen present, the anther geometry and the excitation velocity, that decays over time with a nonlinear decrease in pollen expulsion. Adding inelastic collisions would further slow the rate of pollen release.

Utilized by Hansen *et al.* [23], the billiards model offers additional insights into pollen release by directly calculating particle trajectory from particle–wall collisions. Controlling both the frequency(ies) and amplitude(s) of the anther motion allows for a more granular investigation into the energy applied to the system. This model neglected collisions between pollen particles by assuming that the relative size of the particles is very small with respect to the anther volume and therefore the probability of interaction is likewise small. This assumption tacitly implies that increasing the number of particles in the anther has no effect on pollen expulsion. In addition, since the applied motion was side-to-side, the particles are seeded with an initial, random velocity to ensure they move along the length of the anther. Over the range of frequencies and anther displacements simulated, all but the lowest peak anther velocities saw 90% or more of the particles leave the anther in 0.5 s, demonstrating again a direct relationship between the movement of the system to pollen expulsion.

An alternative to the previous approaches, the discrete element method (DEM) has seen renewed interest due to the relatively easy access to high-performance computers and the ease with which the algorithm can be parallelized. DEM is able to simulate millions of particles simultaneously and the number of particles is only limited by the computational resources available. Similar to the billiards model, particle trajectories are solved through time. However, instead of being governed by reflections, the acceleration of DEM particles is calculated from the summed interactions with each other, wall boundaries and additional external forces. DEM has been used to model granular solids to determine quantities too difficult to measure experimentally, such as the values and distributions of forces in spherical packing [24] and the self-assembly of spheres under one-dimensional vibrations [25]. DEM is a flexible tool that allows for a broader range of adjustable particle properties and conditions, such as non-uniform particle shapes and cohesive forces that can be broken, necessary for an expansive range of future work.

This paper leverages DEM to simulate and analyse the pollen–pollen and pollen–wall interactions in a vibrating anther, and subsequently, the rate of pollen expulsion. The results from a parameter sweep of frequencies and anther displacements demonstrate how DEM relaxes some of the assumptions that have been necessary to model pollen to date.

## 2. The discrete element method

Unlike many traditional modelling approaches that rely on a fixed mesh—a collection of discrete elements that subdivide a domain into smaller and definable pieces to solve partial differential equations—DEM centres the equations of motion on each particle [26]. The collection of surface and body forces acting on a particle of mass m are summed to find the total force, which then solves for that particle’s acceleration according to Newton’s Second Law (${𝐅}_{𝐭𝐨𝐭}=m𝐚$). The balance of rotational momentum is also considered in DEM, but by assuming pollen to be smooth, spherical grains [27], the rotational energy is vanishingly small in this problem and a detailed description is therefore ignored. With a small enough time step, Δt, the position, 𝐱, of every particle is updated by first updating each particle’s velocity through ${𝐯}_{𝐧𝐞𝐰}={𝐯}_{𝐨𝐥𝐝}+𝐚\Delta t$, and then its position ${𝐱}_{𝐧𝐞𝐰}={𝐱}_{𝐨𝐥𝐝}+{𝐯}_{𝐧𝐞𝐰}\Delta t$. The process repeats over all particles until the simulation ends. Bold variables indicate vector quantities. The mesh-free DEM model in Siemens’ SimCenter STAR-CCM + 2206 [28] was utilized for this study.

As the anther was translated side-to-side, the number of pollen–pollen and pollen–wall collisions and the pollen released from the anther were tabulated. Though not leveraged in this study, DEM also allows particle position data to be collected over the entire duration of a simulation, which can be used to determine individual particle velocities and accelerations, and also aggregate data, such as the mean number of collisions before an individual pollen particle is released or the characteristic trends for pollen given a distribution of sizes.

Aerodynamic forces act on the surface of a particle and are caused through interactions with the surrounding fluid. These consist of the drag force from viscous shears and pressure distributions caused by moving in a fluid, the force from an additional pressure gradient, like buoyancy, and the added mass force, which is a contribution to the inertia of an accelerating or decelerating object as it displaces a fluid. In [22], aerodynamic forces had little to no impact on the amount of pollen expelled, but did reduce the distance travelled once outside the anther. Because the study only considers pollen inside the anther, we assume the pollen particles are travelling within a vacuum and, by extension, all surface forces cancel.

The body forces acting on the particles consist of the force from gravity, contact forces described by the chosen contact model and the Coulomb force that accounts for electrostatic attraction or repulsion. A study by Bowker & Crenshaw [29] found an average positive charge across seven plant species of approximately 0.32 fC, with values ranging from −0.6 to 1.2 fC depending on the species, yielding electrostatic forces that are of the same order as the gravitational force. The anther wall may shield pollen from electrostatic forces [29], but contactless transport has been observed from flowers lacking poricidal anthers to electrostatically charged butterflies and moths [30]. From preliminary simulations, it was determined that gravitational acceleration had a negligible impact and mirrors the observations of Buchmann & Hurley [22] where the anther orientation did not significantly impact pollen expulsion. Although Nevard and Vallejo-Marín examined pollen deposition rather than expulsion, they similarly found no significant effect of floral orientation on deposition across multiple *Solanum* species [31]. As such, gravity and electrostatic forces are also excluded in this initial study. Considering these simplifications, the total force acting on a pollen particle consists of only contact forces with other particles and walls.

## 3. Contact forces

**Figure 1.**
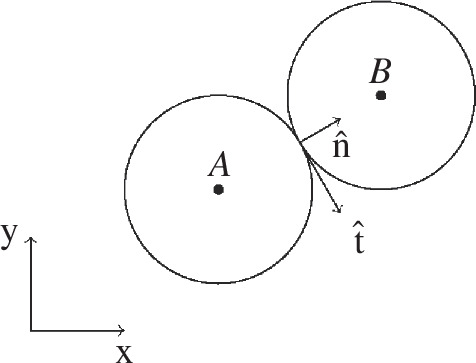
Simple collision of two particles with tangential and normal force components.

The Hertz–Mindlin no-slip contact model is used to calculate the normal, $F_{n}$, and tangential, $F_{t}$, components of the reaction force from two colliding particles


(3.1) $$F_{c}=F_{n}\hat{n}+F_{t}\hat{t},$$

where the normal direction, $\hat{𝐧}$, follows the line connecting their centres and the tangential direction, $\hat{𝐭}$, is parallel to a modified difference of the two particle velocities [32,33]. Figure 1 shows the contact force between two spheres, A and B. If multiple spheres interact at the same time, the force from each sphere pair is summed into a cumulative contact force.

The normal and tangential force components are calculated using equations (3.2*a*)–(3.2*c*) and equations (3.3*a*)–(3.3*c*), respectively, with the forces combining the response from the material stiffness, K, and damping, N. Stiffness quantifies the force required to stretch or compress a particle by a unit length and damping refers to the force that opposes how quickly this occurs, scaled by the coefficient of restitution, $C_{rest}$. This coefficient is a non-dimensional number that represents the fractional amount of kinetic energy that remains after a collision, with $C_{rest}=1$ being perfectly elastic, and all the energy being absorbed when $C_{rest}=0$. This is seen as the material damping terms vanish when collisions are perfectly elastic. The tangential force is conditionally dependent on the static friction coefficient $C_{fs}$.


(3.2*a*) $$\begin{array}{rl} F_{n} & =-K_{n}d_{n}-N_{n}v_{n}, \end{array}$$

(3.2*b*) $$\begin{array}{rl} K_{n} & =\frac{4}{3}E_{eq}\sqrt{d_{n}R_{eq}}, \end{array}$$

(3.2*c*) $$\begin{array}{rl} N_{n} & =\frac{-\sqrt{\left(5K_{n}M_{eq}\right)}\ln\left(C_{n\;rest}\right)}{\sqrt{\pi^{2}+\ln{\left(C_{n\;rest}\right)}^{2}}}. \end{array}$$

Because of the finite scale of the time step, the geometry of the two particles will intersect by a small amount, d, in each direction. The relative velocity between the two particles, 𝒗, is split into normal and tangential components, with the latter deriving the tangential direction $\hat{𝐭}$. The subscripts n and t denote normal and tangential components, respectively, for any of the terms above.


(3.3*a*) $$\begin{array}{rl} F_{t} & =\left\{ \begin{array}{ll} \frac{|K_{n}d_{n}|C_{fs}d_{t}}{|d_{t}|}, & |K_{t}d_{t}|<|K_{n}d_{n}|C_{fs},\\ -K_{t}d_{t}-N_{t}v_{t} & \text{otherwise}, \end{array}\right. \end{array}$$

(3.3*b*) $$\begin{array}{rl} K_{t} & =8G_{eq}\sqrt{d_{n}R_{eq}}, \end{array}$$

(3.3*c*) $$\begin{array}{rl} N_{t} & =\frac{-\sqrt{\left(5K_{t}M_{eq}\right)}\ln\left(C_{t\;rest}\right)}{\sqrt{\pi^{2}+\ln{\left(C_{t\;rest}\right)}^{2}}}. \end{array}$$

Equations (3.2) and (3.3) utilize equivalent physical values described by equations (3.4*b*)–(3.4*d*),


(3.4*a*) $$\begin{array}{rl} M_{eq} & =\frac{1}{\frac{1}{M_{A}}+\frac{1}{M_{B}}}, \end{array}$$

(3.4*b*) $$\begin{array}{rl} R_{eq} & =\frac{1}{\frac{1}{R_{A}}+\frac{1}{R_{B}}}, \end{array}$$

(3.4*c*) $$\begin{array}{rl} E_{eq} & =\frac{1}{\frac{1-\nu_{A}^{2}}{E_{A}}+\frac{1-\nu_{B}^{2}}{E_{B}}}, \end{array}$$

(3.4*d*) $$\begin{array}{rl} G_{eq} & =\frac{1}{\frac{2(2-\nu_{A})(1+\nu_{A})}{E_{A}}+\frac{2(2-\nu_{B})(1+\nu_{B})}{E_{B}}}, \end{array}$$

where sphere A and sphere B can have distinct mass M, radii R, and material properties, E is the Young’s modulus and ν the Poisson’s ratio. The shear modulus, G, can be calculated from the others. When applying the Hertz–Mindlin no-slip contact model to a sphere and a wall, the wall radius and mass are assumed to be infinite $R_{wall}=\infty$ and $M_{wall}=\infty$.

## 4. The simulation environment

**Figure 2.**
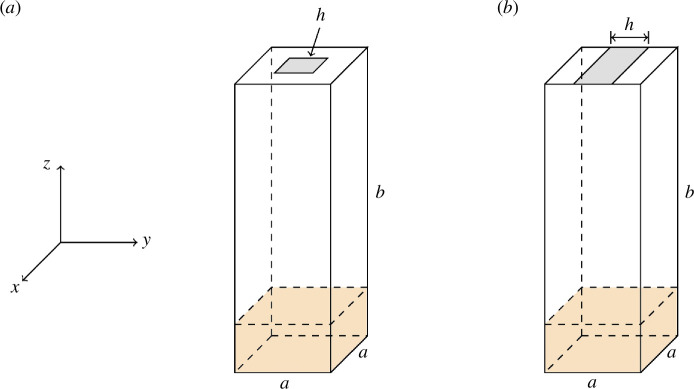
Poricidal geometry representation (*a*) and pseudoporicidal geometry representation (*b*). The orange shaded volume represents the region where particles are injected and the grey shaded area represents the pore exit. These geometries are approximations for anthers seen in the tomato flower.

Simplifying the anther to a box, two geometries were created from the approximation of *S. lycopersicum*, Solanaceae in [23]: a pseudoporicidal anther modelled as the extruded two-dimensional shape in [23], resulting in a slit at the apical end, and a poricidal design, where a finite-sized pore is placed at the apical end, better representing a real pore [22]. Both anther geometries were defined with width and length a=0.69mm and height b=5.07mm. Additionally, a hole at the top of the anther was dimensioned with h=0.22mm, such that the slit of the pseudoporicidal model accounted for 31.9% and the poricidal model was a square occupying 10.2% of the total top area. The anther geometry and pore shapes can be seen in figure 2.

Modelled after the vibration profile of [23], the anther is subjected to a strictly translational motion along the *y*-axis of figure 2, in which the position of the anther wall is described by equation (4.1),


(4.1) $$S_{wall}=\eta (\cos(\omega_{1}t)+\cos(\omega_{2}t))$$

and subsequently the velocity of the anther wall is described by equation (4.2),


(4.2) $$v_{wall}=-\eta (\omega_{1}\sin(\omega_{1}t)+\omega_{2}\sin(\omega_{2}t)),$$

where t represents time and η is the half-peak displacement amplitude. Because of the two components in $S_{wall}$, the peak displacement amplitude is 2η and ranged from 0.4 to 2 mm in increments of 0.4 mm. Additionally, $\omega_{1}$ is the varied frequency and $\omega_{2}$ is a stationary frequency that was maintained at 390 Hz. The varied frequencies were 150, 200, 300 and 400 Hz (capturing the buzzing range) and from 550 to 1600 Hz in increments of 350 Hz. This large range of frequencies was chosen to provide a direct comparison with the work done in [14,23] and to discern if modelling provided any insight on the impact of frequency on pollen release. Contrary to [23], a fixed $\omega_{2}$ does not guarantee a beat or pulse of the variable frequency, but it does create a more organic signal than what would be constructed from a pure tone. Though these excitation frequencies extend above the approximate 100–400 Hz buzzing frequency range observed in bees, we note that potential artificial pollination technologies are not subject to the same frequency constraints as biological pollinators. It is therefore of interest to explore excitation frequencies outside the biological range to understand if these provide value in terms of pollen expulsion.

In each of the 80 simulations, 10 000 pollen particles were randomly seeded in the bottom 5% of the anther and represented by the shaded region in figure 2. The number of pollen in an anther can vary wildly across plant species, but Kemp and Vallejo-Marín observed averages between 22 000 and 466 000 grains for six species of *Solanum* [17], Vallejo-Marín *et al.* observed averages between 29 000 and 800 000 for six species of *Solanum* [34], and Harder and Barclay an average of 1 270 700 pollen grains for experiments with *Dodecatheon conjugens* [15]. In choosing our number, we compared simulations with 10 000 and 100 000 particles and did not find a noticeable difference across our analyses to warrant the increased computational time. The particles were seeded in the bottom of the anther with the assumption that pollen can be agitated and settle in a common area, resulting in a packing density of 34.7%. Lower packing densities would result in fewer particle collisions at the start of the simulation, slowing the rate of pollen release. The coefficients of restitution were chosen to represent a perfectly elastic system and provide the most direct comparison with the billiards model. The pollen particles started with a zero initial velocity and properties given in table 1. The Young’s modulus of pollen varies based on the hydration of the individual particle, and an approximate value for pollen was selected based on the work of Qu & Meredith [37]. The density of pollen also varies based on the hydration of the individual pollen particle [38], with studies showing densities between 968 and 2238 kg m^−3^ [39]. For the purposes of having an agnostic model, 1000 kg m^−3^ was chosen as a round number within this range. It is important to note that under perfectly elastic conditions, the Young’s modulus merely acts as a scaling factor for how quickly a pollen particle responds to a collision and the particle mass is ignored. Simulations ran for 0.5 s with a time step of ∆t = 1 µs.

**Table 1.** Pollen properties [35]${\;}^{*}$, [36]${,}^{†}$, [37]${,}^{‡}$ and simulation values^§^.

variable	value
$E^{‡}$	1.6 GPa
$\nu^{*}$	0.45
$\rho^{§}$	1000 kg m^−3^
$R^{†}$	0.01 mm
$C_{n\;{rest}^{§}}$	1.0
$C_{t\;{rest}^{§}}$	1.0
$v_{0^{§}}$	0 m s^−1^

## 5. Results and discussion

For all the conditions simulated, particle expulsion of 99% or more was achieved within 0.5 s. These results are in good agreement with the billiards model, except at the lowest frequency and amplitude combinations where the number of pollen released falls off rapidly and is closer to 40–50% at 400 Hz and 0.2 mm peak displacement. This smaller percentage is closer to the amount expelled (57.8%) in experiments of *Bombus impatiens* during their first visit to a tomato flower [40]. However, the billiards model ignores particle–particle interactions, which account for a significant number of the total collisions and are anticipated to increase the amount of pollen released. The statistical mechanics model estimates that 90% of the pollen is released in 0.0128 s at 400 Hz and 0.4 mm displacement for this geometry, and increases to 0.192 s when the coefficient of restitution is reduced to $C_{rest}=0.1$. Furthermore, all of the modelled expulsion rates are larger than experimental results. Kemp and Vallejo-Marín show 3−10 s of continuous buzzing is necessary to release all available pollen across six *Solanum* section *Androceras* species [17]. An order of magnitude difference in expulsion time is significant; however, as demonstrated in [22], the inclusion of material damping drastically slows pollen release of this order and is probably the most significant contributor to the differences seen between the current DEM model and the times measured in experiments and natural observations. Further, as demonstrated in these results and in [17], the pore geometry and size are also factors.

**Figure 3.**
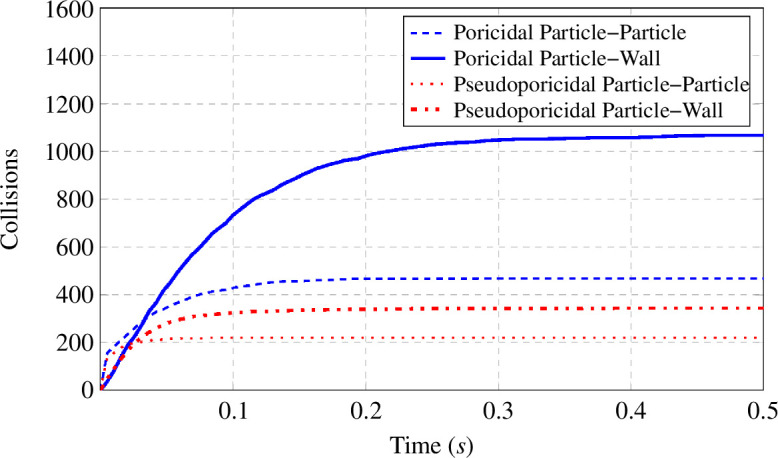
Comparison of cumulative particle interactions between poricidal and pseudoporicidal systems during the solution time of 0.5 s.

Starting from rest, particles are pushed by the wall in the direction of the wall motion, $S_{wall}$, but quickly transition to collisions with one another. These particle–particle collisions rapidly diffuse the kinetic energy within the anther and introduce a velocity component that is no longer aligned with $S_{wall}$, allowing particles to move towards the exit. After a short period of time, the distance between particles is sufficiently large that particle–wall interactions become dominant. As the net energy within the anther increases, pollen particles begin to escape the anther. Then, as a consequence of both the distance between particles and particles leaving through the exit, the number of new particle–particle collisions asymptotes to zero first. Finally, the particle–wall collisions continue until the anther empties. This pattern is observed across all simulations and aligns with the theoretical considerations outlined by Buchmann & Hurley [22]. The pseudoporicidal exit shows a lower total number of interactions and is probably due to the larger available exit area, which reduces the chance of a particle bouncing back towards the base of the anther. An example of the total number of particle–particle and particle–wall collisions is shown in figure 3 for η=0.4 mm and $\omega_{1}=150$ Hz. Interestingly, particle–particle collisions accounted for an average across the parameter sweep of 30.03±1.32% and 39.50±2.67% of all collisions in poricidal and pseudoporicidal anthers, respectively.

It is suspected that the large increase in the amount of pollen expelled versus the ranges observed in experimental work is principally a result of the neglected material damping present within anther walls and pollen particles and aerodynamic drag. As derived in [22], the restitution coefficient plays a significant role in the available kinetic energy within the anther, where setting $C_{rest}=0.1$ has a three orders-of-magnitude reduction in the normalized kinetic energy versus the perfectly elastic system. While values of $C_{rest}$ can be calibrated in future efforts and when a specific species is to be modelled, to provide a better fit between models and the physical world, an agnostic model has been presented to understand broader trends that can be applied across anther and pollen geometry and properties, and buzzing dynamics. Additional losses, such as the break-up of pollen clusters and aerodynamic loads, would further reduce the number of pollen released over a given period of time.

**Figure 4.**
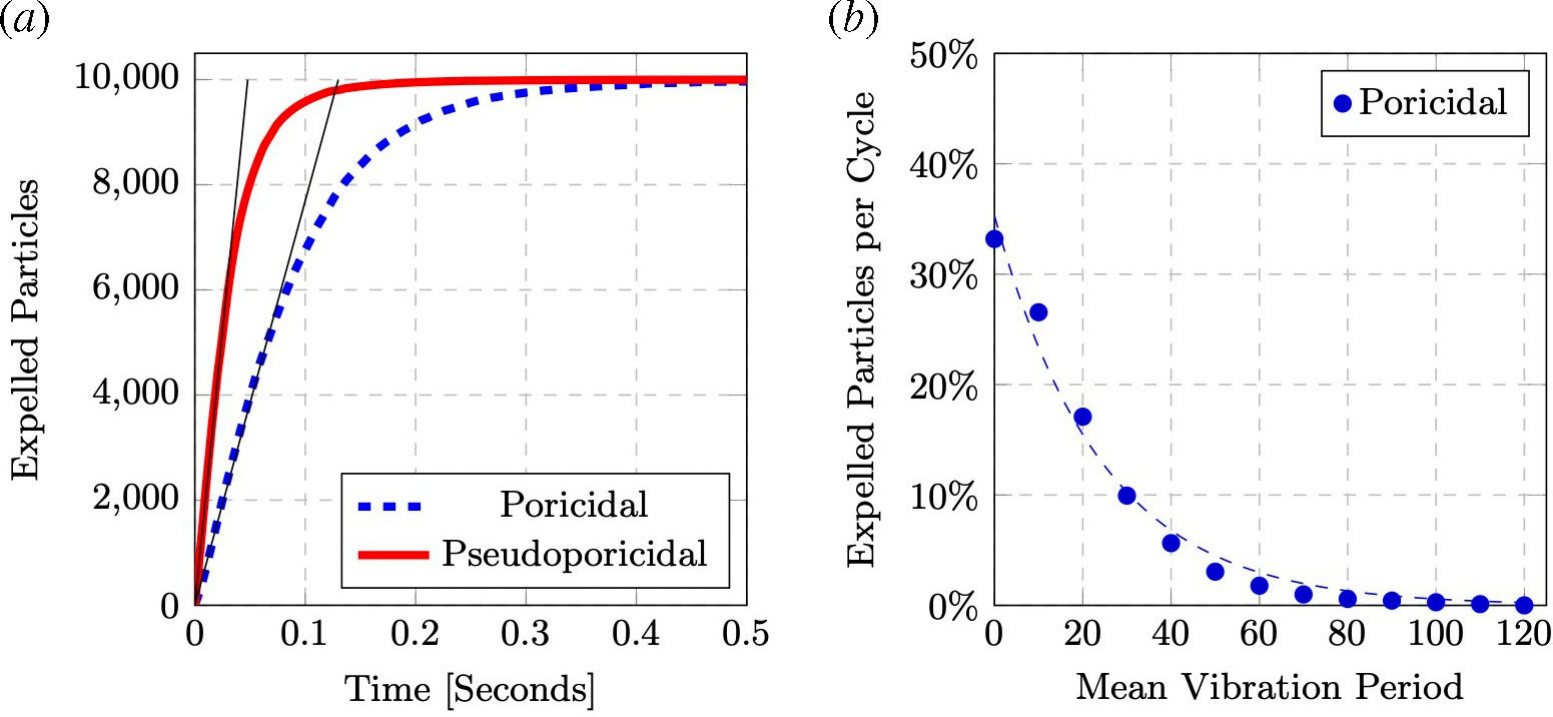
(*a*) Examples of both poricidal and pseudoporicidal geometries pollen expulsion at η=0.4 mm and $\omega_{1}=150\;Hz$ seeded with 10 000 particles. The thin lines designate the initial expulsion rate of the particles, *Ṗ*, seen in figure 5. (*b*). The per cent of total pollen particles removed from the anther per mean vibration period, where the dashed line represents the line of best fit: $y=35.2891(1-0.0403{)}^{x}$

The increase and decrease in kinetic energy are also captured in the total number of particles expelled from the anther over time, with a representative time-history shown in figure 4*a*. For both the poricidal and pseudoporicidal geometries, particles are released faster when more particles are present and slower as fewer and fewer particles are present. Whether a result of the reduced kinetic energy available with a smaller number of particles or their distribution throughout the entire anther at later times, this slowing of particle release is observed through smaller dosings with repeated visits [17,40]. Though unsurprising, larger openings are also less restrictive and allow more particles to leave sooner.

An alternative way to consider pollen expulsion over time is the percentage of the total original pollen released during a vibration period, and shown to match an exponential decay across six *Solanum* species [17]. Figure 4*b* shows that the expelled pollen over time matches this expected decay and that they provide an equivalent, while still different, vantage to the data. The work of Kemp & Vallejo‐Marín [17] found dispensing rates between 5 and 87% across the different species and applied velocities per vibration, which was defined as five 200 ms pulses at 300 Hz. It is difficult to define a similar ‘vibration’ for a signal with multiple frequencies and we have therefore chosen a single period of the mean frequency, $\omega_{m}=\sqrt{\omega_{1}\omega_{2}}$. Our dispensing rate of 4% actually fits in fewer total oscillations of a single vibration as defined in [17]. In addition to the suspected causes above, the rapid pollen release is also related to our smallest velocity (678.6 mm s^−1^) being an order of magnitude larger than their experiments (80 mm s^−1^).

**Figure 5.**
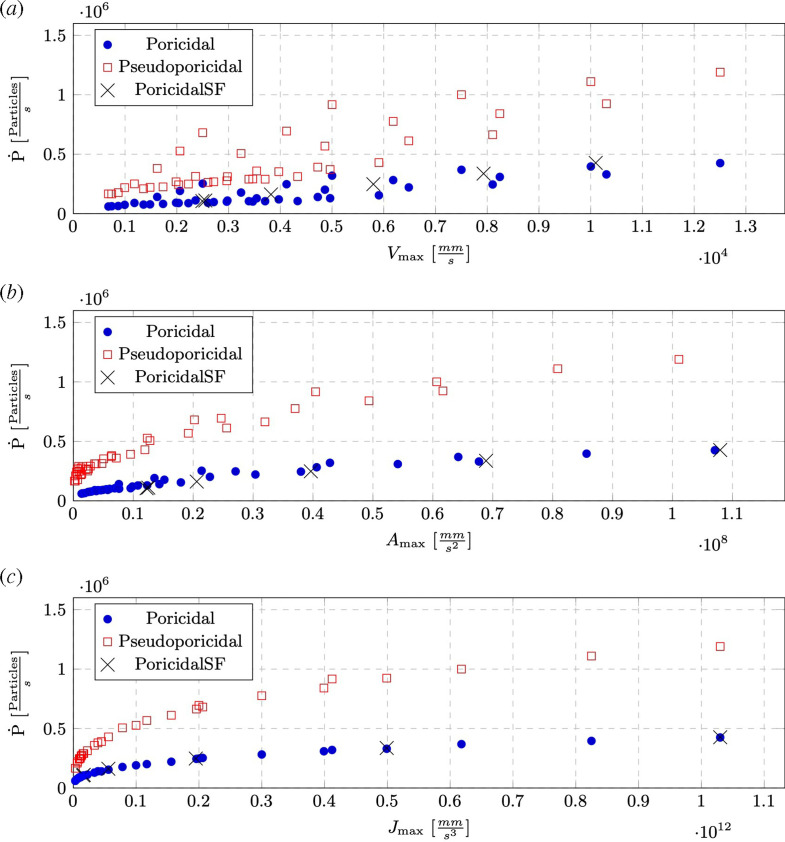
The initial pollen expulsion rate for both poricidal and pseudoporicidal anthers as a function of the maximum velocity (*a*), acceleration (*b*) and jerk (*c*) of the anther walls. Poricidal SF indicates points where a different vibration profile, $S_{wall}=\eta \cos(\omega_{1}t)$, was utilized to test the effect of a single frequency.

While there was no difference in the amount of pollen released over the 0.5 s of each simulation, a linear trend of the number of particles released over time was consistently observed. This initial pollen expulsion rate, *Ṗ,* with units particles s^−1^ and seen as the thin, straight lines in figure 4, varied across the different parameter combinations, with the smallest rate corresponding to the smallest frequency and amplitude pair and the largest rates with the largest parameter combinations seen in figure 5. *Ṗ* is the slope of this line, and across these simulations evaluated between the start of the simulation and when half of the seeded particles escaped the anther.

In discussing anther dynamics, only displacement amplitude and frequency are independent variables, i.e. the velocity (equation (4.2)) and acceleration are time derivatives of the displacement profile and intrinsically coupled to both η and ω. Rather than considering these variables at odds with each other, all of the time derivatives of displacement collapse into a single variable. Here, we introduce the maximum jerk, $J_{max}=\eta (\omega_{1}^{3}+\omega_{2}^{3})$, to describe how vigorously the anther shakes, or the change in acceleration over time, where jerk is defined as the third derivative of position x(t) with respect to time: $J(t)=\frac{d^{3}x}{dt^{3}}$. A comparison of the initial pollen expulsion rate against the maximum velocity, acceleration and jerk of the anther walls is shown in figure 5 for all simulations. While these results show a direct increase in pollen expulsion across the definitions of vibration amplitude, the data are seen to collapse most consistently when using maximum jerk as opposed to the other time derivatives. This suggests that it does not matter whether frequency or amplitude drives this increase and that there are diminishing returns to how quickly pollen is released as the anther is shaken more. The results also clearly show that *Ṗ* slows as $J_{max}$ reaches significantly large values. Moreover, five additional simulations were run with a single, continuous frequency with the poricidal geometry and the results fall right on top of these curves (shown as black Xs in figure 5). The implication is that, for purely translational motions, the specifics of how an anther is shaken are less important than how aggressively. It is also important to note that the starting density of pollen will have a direct impact on *Ṗ*. As the packing density is decreased, *Ṗ* will also decrease. For simulations of prismatic geometry with smooth walls (the cross section is unimportant) and a purely translational motion, this will asymptote to zero as the pollen particles are spaced so far apart they only collide with the wall and no longitudinal motion is imparted to move towards the anther pore.

## 6. Conclusions

This research has utilized the discrete element method to begin exploring the complex dynamics of pollen within flower anthers during buzz pollination, subjected to simple translating oscillations. Our findings reveal a strong correlation between the maximum jerk, $J_{max}$, of anther walls and the initial rate of pollen expulsion. The simulations indicate that while increased rates of expulsion are achievable across a spectrum of values, further increases in vibration intensity show diminishing returns. This relationship supports the existing literature that an ‘optimal’ vibration frequency may not exist, at least if the pollen motion is modelled independently from the structural dynamics of the anther. Future simulations should explicitly couple the anther’s structural dynamics to pollen modelling to better address if anther resonance increases pollen expulsion rates. The simulations also show that by including particle–particle interactions, we are able to expel pollen starting at rest using a purely transverse motion. Furthermore, approximately one-third of collisions that occurred within the anthers were between particles, contradicting previous estimates that particle–particle collisions were unimportant. Particle–particle collisions were seen to be especially important at the start of the vibration, with diminishing influence over time. Differences between the poricidal and pseudoporicidal anther exits indicate a dependence on the pore size and shape when quantifying the pollen expulsion rate.

This method of modelling also leaves room for including currently omitted factors, such as cohesion/adhesion and Coulomb forces, with only minor changes to the current approach. Further investigations could extend the range of modelled anther and pollen types and refine predictive models based on these findings. Additionally, expanding the variety of anther geometries examined could provide deeper insights into the mechanical interactions within buzz pollination.

## Data Availability

The data supporting this study on pollen expulsion dynamics in vibrating anthers are publicly available in the Dryad Digital Repository [41]. The dataset includes raw simulation data of particle counts, particle–particle, and particle–wall collisions over time across the parameter sweep of frequencies and amplitudes. MATLAB scripts for data analysis are included to assist in reproducing the study’s results and visualizations. A template simulation file (*.sim) for η = 0.4 mm and ω_1_ = 150 Hz can be found in the electronic supplementary material at [42].
